# Monodisperse nanosheet mesophases

**DOI:** 10.1126/sciadv.adk6452

**Published:** 2024-06-05

**Authors:** Nobuyoshi Miyamoto, Momoka Miyoshi, Riki Kato, Yuji Nakashima, Hiroyuki Iwano, Takashi Kato

**Affiliations:** ^1^ Department of Life, Environment and Applied Chemistry, Faculty of Engineering, Fukuoka Institute of Technology, 3-30-1, Wajiro-Higashi, Higashiku, Fukuoka 811-0295, Japan.; ^2^ Department of Life, Environment and Applied Chemistry, Graduate School of Engineering, Fukuoka Institute of Technology, 3-30-1, Wajiro-Higashi, Higashiku, Fukuoka 811-0295, Japan.; ^3^ Department of Chemistry and Biotechnology, School of Engineering, The University of Tokyo, 7-3-1 Hongo, Bunkyo-ku, Tokyo 113-8656, Japan.; ^4^ Research Initiative for Supra-Materials, Shinshu University, 4-17-1, Wakasato, Nagano 380-8553, Japan.

## Abstract

Self-assemblies of anisotropic colloidal particles into colloidal liquid crystals and well-defined superlattices are of great interest for hierarchical nanofabrications that are applicable for various functional materials. Inorganic nanosheets obtained by exfoliation of layered crystals have been highlighted as the intriguing colloidal units; however, the size polydispersity of the nanosheets has been preventing precise design of the assembled structures and their functions. Here, we demonstrate that the anionic titanate nanosheets with monodisperse size reversibly form very unusual superstructured mesophases through finely tunable weak attractive interactions between the nanosheets. Transmission electron microscopy, polarizing optical microscopy, small-angle x-ray scattering, and confocal laser scanning microscopy clarified the reversible formation of the mesophases (columnar nanofibers, columnar nematic liquid crystals, and columnar nanofiber bundles) as controlled by counter cations, nanosheet concentration, solvent, and temperature.

## Teaser

Size-regulated inorganic nanosheets were assembled into unusual columnar nanofibers and superstructured mesophases.

## INTRODUCTION

Self-assemblies of anisotropic colloidal particles into colloidal liquid crystalline (LC) phases (*1*–*13*) and well-defined one-dimensional (1D) (*14*–*21*), 2D (*22*–*25*), and 3D (*26*–*28*) superlattices are of great interest for hierarchical nanofabrications applicable for electric, optical, catalytic, mechanical, ion/molecular transport, and energy functional materials (*29*–*34*). Among various anisotropic particles, inorganic nanosheets obtained by exfoliation of layered crystals have been recently highlighted (*35*) because of the diversities of the materials and functions (*36*, *37*) as well as spontaneous formation of mesophases. However, the large polydispersity of lateral size and irregular shapes of the nanosheets have been preventing precise design of the mesophases and functions: The mesophases of the polydisperse nanosheets are mostly limited to nematic (*5*–*8*, *11*) and swollen lamellar LC phases (*3*, *12*) in repulsion-dominated systems regulated by entropic interactions (*38*). Although a few cases of assembling size-regulated nanoplates into liquid crystals (*2*) and 1D superlattices (*14*–*21*, *39*) were reported, the structures were irreversibly formed as nonequilibrium states so that fine structural control was difficult. Hierarchical structure design and material diversity for applications also remain as great challenges.

Here, we demonstrate that monodispersed titanate nanosheets (mNSs) reversibly form highly regulated superstructured mesophases through finely tunable weak attractive interactions and entropic interactions. Transmission electron microscopy (TEM), polarizing optical microscopy (POM), small-angle x-ray scattering (SAXS), and confocal laser scanning microscopy (CLSM) clarified the reversible formations of the mesophases, columnar nanofibers (ColNFs), columnar nematic liquid crystals (ColNF-Nems), and ColNF bundles (ColNF-Buns), as controlled by counter cation concentration, nanosheet concentration, solvent, and temperature. The mesophases (nematic and columnar LC phases) of monodisperse gibbsite plates were reported before (*2*), which were driven solely by entropic interactions among the particles in the absence of attractive interactions; however, ColNF-Nem has never been reported. In addition, compared to previous monodisperse nanoplatelet systems that were assembled as superlattices (*14*–*21*), the present titanate mNSs are the only anionic system so that the material design by the combination with cationic functional species is possible.

## RESULTS

**Fig. 1.**
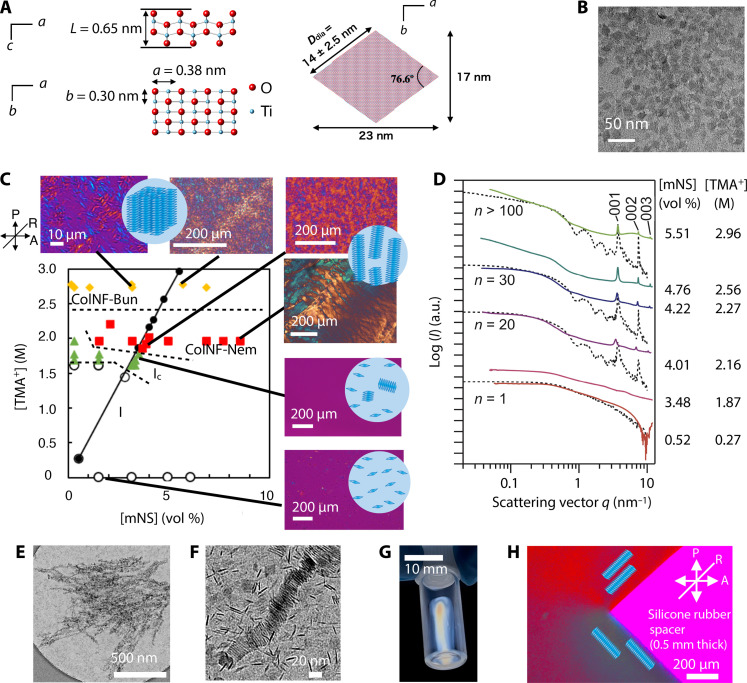
Formation of ColNFs, ColNF-Nem, and ColNF-Buns of the mNSs controlled by ionic strength and nanosheet concentration. (**A**) Schematic structure of the mNS and (**B**) its TEM image. (**C**) The phase diagram, POM images, and schematic structures of the mNS aqueous dispersions as the function of [mNS] and [TMA^+^]. The labels I and Ic represent isotropic dispersions of the mNS and ColNF, respectively. (**D**) SAXS patterns of the mNS dispersions with varied [mNS] and [TMA^+^], which are also indicated as black filled triangle plots in the phase diagram. The dashed lines are the simulated SAXS curves for the ColNFs with *n* layers (figs. S4 and S5 for details). a.u., arbitrary units. (**E**) TEM and (**F**) cryo-TEM images of the ColNFs. (**G**) The observation under crossed polarizers of the ColNF-Nem sample ([mNS] = 3.1 vol % and [TMA^+^] = 2.2 M) in a glass vial and (**H**) its POM image at the interface between the colloidal sample and the spacer (silicone rubber sheet with the thickness of 0.5 mm). The arrows A and P show the directions of analyzer and polarizer, respectively. The line R is the direction of the λ plate with the retardation of 530 nm.

We synthesized the mNSs by the bottom-up method (*40*, *41*) instead of the conventional exfoliation method (*42*). The mNSs obtained in this study were characterized as single-layer nanosheets of lepidocrocite-type titanate [Ti_1.825_O_4_]^0.7−^ with the diamond-shape and monodisperse side length (lateral size) controllable in the range of 5 to 30 nm. The formation of mNSs with the side length of 14 ± 2.5 nm and the thickness of 0.65 nm (Fig. 1A) was confirmed by TEM (Fig. 1B), x-ray diffraction (XRD) (fig. S1), and SAXS (fig. S2). The as-obtained aqueous colloidal sol containing 0.52 vol % of the mNSs and 0.27 M tetramethylammonium (TMA^+^) was transparent and nonbirefringent. SAXS measurements confirmed the uniform and isotropic dispersion of the mNSs (fig. S3) in the colloidal sol.

In the process of condensing the as-prepared colloidal sol, we found very unusual self-assembling behaviors of the mNSs, which were controlled by [TMA^+^] and [mNS], as summarized in the phase diagram (Fig. 1C): mNSs were self-assembled into isotropically dispersed ColNFs and then into ColNF-Nem of the aligned ColNFs and ColNF-Bun of the assembled ColNFs, depending on [TMA^+^] and [mNS]. In the TEM images of the ColNFs (Fig. 1, E and F), we observe string-like objects with the length of submicrometer and the width of ~20 nm; the width is mostly consistent with the lateral size of the mNS. In the magnified cryo-TEM image, we observe the nanosheets stacked with the periodic distance of 1.7 nm inside the ColNFs. In the SAXS profiles (Fig. 1D, purple and blue lines), with the increase in [TMA^+^] and [mNS], sharp and intense peaks arose at *d* = 1.7, 0.9, and 0.6 nm, which are due to the Bragg diffractions from the stacked structure. The stacking periodicity evaluated by SAXS is consistent with that evaluated by cryo-TEM. Considering the size of hydrated TMA^+^ (1.05 nm in diameter) and the thickness of the mNS (0.65 nm), the hydrated TMA^+^ ions are intercalated in the stacks of the mNSs. In addition, we observed excess scattering in the lower-*q* region of the SAXS profiles, which indicates the presence of larger objects consisting of the mNSs. The observed SAXS curves mostly coincided with the simulated scattering curves of the ColNFs (Fig. 1D, dashed lines, and figs. S4 and S5). Thus, we confirmed the formation of the ColNFs through 1D stacking of the mNSs.

With the increase in [TMA^+^] and [mNS], more ColNFs formed and grew longer, leading to the formation of the LC phase (ColNF-Nem). The formation of this LC phase was confirmed by permanent birefringence observed with the naked eye under crossed polarizers (Fig. 1G) and by the textures characteristic to ColNF-Nem phase observed by POM (Fig. 1C) (*43*). In the POM observation at the spacer/liquid interface parallel and perpendicular to the slow axis of the retardation plate (Fig. 1H), orange and blue interference colors are observed, respectively. This indicates that the mNS plane is preferentially aligned perpendicular to the interface, in contrast with the conventional LC nanosheets (*5*) that align parallel to the interface (see fig. S6 for details). This again confirms the formation of the ColNFs because such the unusual alignment of the nanosheets perpendicular to the interface is explained by the preferential orientation of the ColNFs along the interface. Although a few cases of assembling monodisperse nanoplates into stacked ribbons (*18*, *20*) and superlattices (*26*) have been reported, those were formed by tricky solvent evaporation processes as nonequilibrium states and LC phases have never been reported. In contrast, the present system gives stable and reversible superstructures in the equilibrated solution phase so that further precise controls and rational designs are possible with various experimental parameters as described later.

**Fig. 2.**
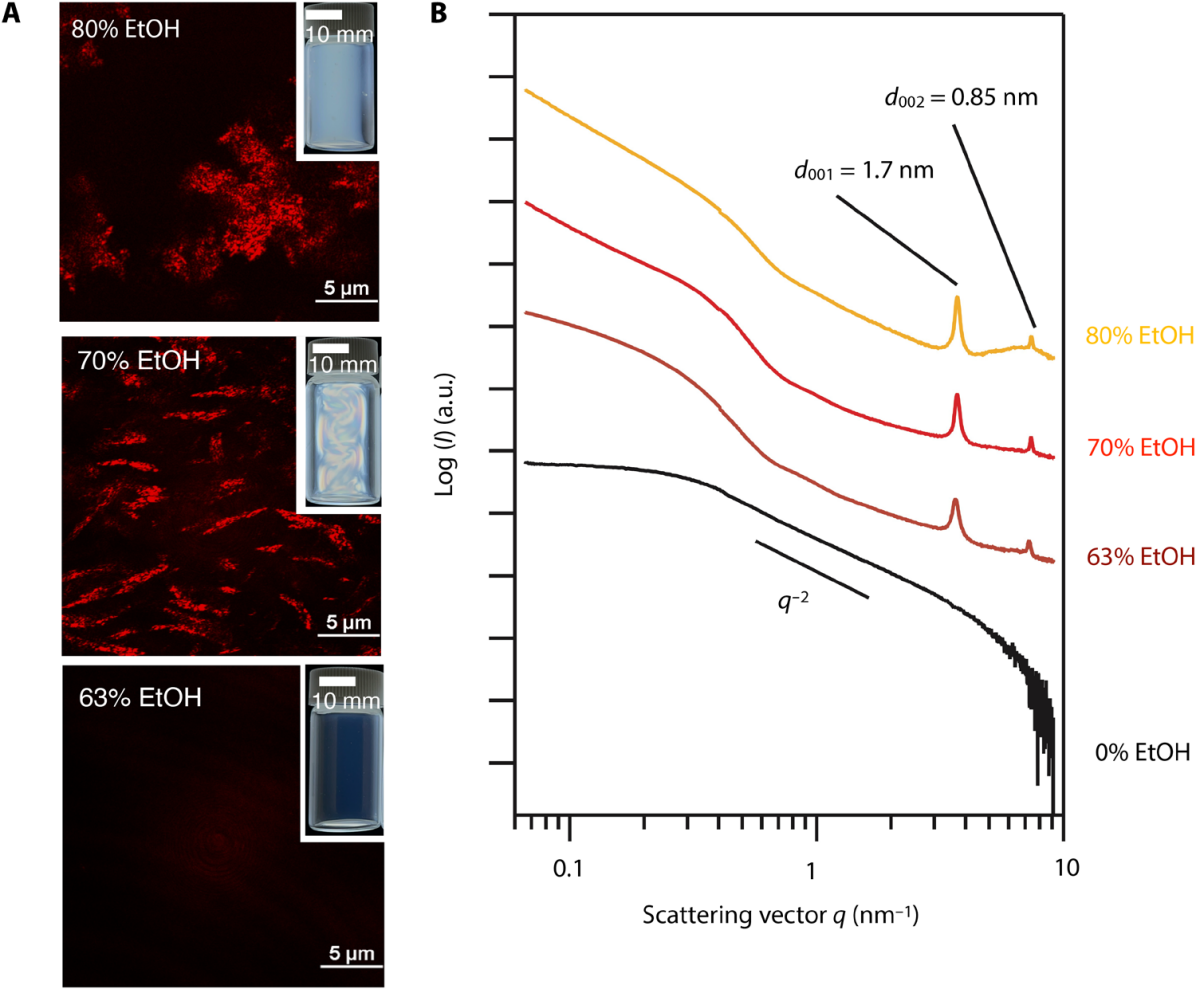
EtOH concentration dependence of the mesophases of the mNS/water/EtOH colloidal sols. (**A**) Photographs under crossed polarizers and CLSM images of the colloidal sols ([TMA^+^] = 0.08 M and [mNS] = 0.15 vol %) and (**B**) their SAXS profiles.

The mesophase formation of the mNSs was also controllable by solvents, which finely modified the mNS-mNS interactions. In the observation under crossed polarizers (Fig. 2A), the mNS/water/ethanol (EtOH) mixtures with 65% or less EtOH were transparent and showed no birefringence, while nothing was observed by CLSM (Fig. 2A). In the SAXS profile (Fig. 2B), the peaks at *d* = 1.7 and 0.9 nm and the excess scattering at low-*q* are observed. These results indicate that the small amounts of ColNFs were formed even at lower [TMA^+^] and [mNS], whereas the ColNFs were uniformly and isotropically dispersed without forming the LC phase. With more than 65% of EtOH, flow birefringence or permanent birefringence appeared, and needle-like objects were observed by CLSM, indicating the formation of the ColNF, ColNF-Bun, and ColNF-Nem. With 80% or more EtOH, large aggregates with irregular shapes were observed by CLSM and birefringence was lost. These EtOH -induced mesophase formation is explained as follows. Because of the lower relative permittivity of EtOH (25 at 20°C) than water (80 at 20°C), the dissociation of TMA^+^ from the anionic mNS surface is hindered, and diffuse layer thickness decreases when EtOH is added, resulting in the attraction between the mNSs and formation of the ColNFs. With too much EtOH, the attraction is too strong so that the mNSs aggregates randomly to form the irregular shape aggregates.

**Fig. 3.**
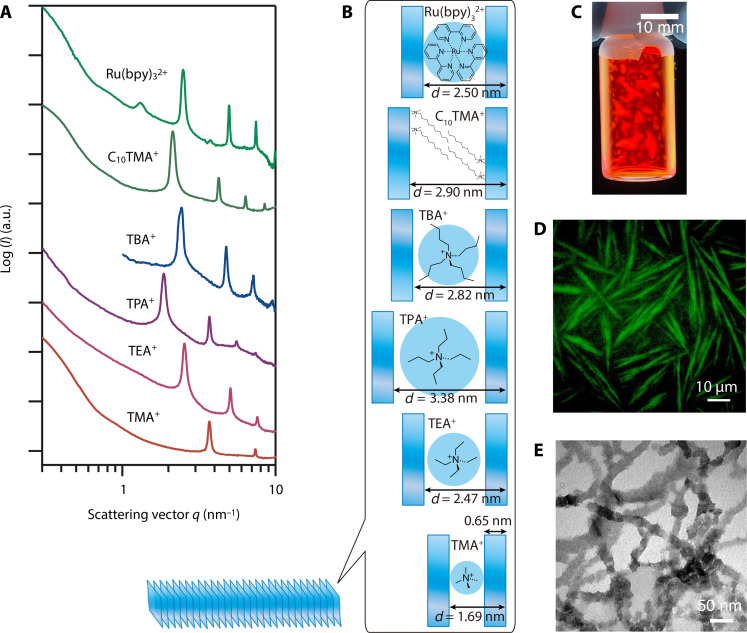
Intercalation of various cationic species into the ColNFs. (**A**) SAXS profiles of the aqueous dispersions of the ColNFs intercalated with TMA^+^, TEA^+^, TPA^+^, TBA^+^, C_10_TMA^+^, and Ru(bpy)_3_^2+^ and (**B**) their schematic structures. (**C**) The photograph under crossed polarizers and (**D**) fluorescence microscopy image of ColNF-Buns intercalated with Ru(bpy)_3_^2+^ ([mNS] = 0.26 vol %, [TMA^+^] = 0.13 M, Ru/CEC = 1.0) and (**E**) TEM image of Ru(bpy)_3_^2+^ intercalated ColNFs (Ru/CEC = 0.8). CEC represents the cation exchange capacity of the mNSs.

For further functionalization of the ColNFs, exchange of the original counter cations of the mNSs, TMA^+^, was very effective. Replacements of TMA^+^ with bulkier tetraethylammonium (TEA^+^), tetrapropylammonium (TPA^+^), tetrabutylammonium (TBA^+^), decyltrimethylammonium (C_10_TMA^+^), or tris(2,2′-bipyridyl)ruthenium(II) [Ru(bpy)_3_^2+^] were successful. In contrast, replacements with inorganic cations such as Na^+^ and Ca^2+^ caused random coagulation of the mNSs due to too strong electrostatic attractions. As shown in Fig. 3 [A (SAXS) and B (schematic drawings)], the interlayer distance increased as the TMA^+^ was replaced with the cations with the larger hydration diameters. All these samples showed birefringent LC phases (Fig. 3C and fig. S7), indicating that the ColNFs and ColNF-Nem are retained even after the cation exchange. In the case of the fluorescent Ru(bpy)_3_^2+^, needle-like objects were observed also by fluorescence microscopy (Fig. 3D) and by TEM (Fig. 3E), confirming that the Ru(bpy)_3_^2+^ is incorporated in the ColNFs and ColNF-Buns. As shown in fig. S8, the fluorescence maximum wavelength for the Ru(bpy)_3_^2+^/mNS system was 599 nm, which was slightly red-shifted from that of the Ru(bpy)_3_^2+^ aqueous solution (589 nm), again confirming the incorporation of Ru(bpy)_3_^2+^ between the mNSs (*44*).

**Fig. 4.**
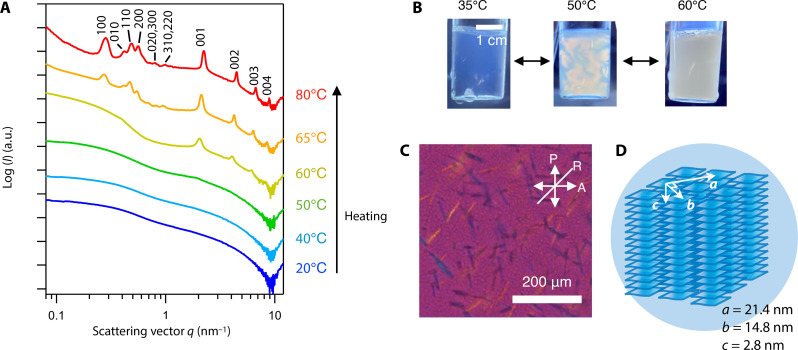
Temperature-controlled mesophase formation of the TBA^+^/mNS aqueous colloid. (**A**) SAXS patterns in the heating process, (**B**) photographs under crossed polarizers, (**C**) POM image at 60°C, and (**D**) schematic structure of the supercrystalline ColNF-Bun. [mNS] = 0.52 vol % and [TBA^+^] = 0.27 M.

We lastly found that the mesophase formation is also controllable by an external factor, temperature, as observed by SAXS (Fig. 4A) and by crossed polarizers (Fig. 4B). At 20°C in the TBA^+^ system, the sample was isotropic, transparent, and not birefringent; no peaks due to stacked nanosheets were observed in SAXS profile. At 50°C, flow birefringence appeared, and the peak at *d* = 2.84 nm and excess scattering in low-*q* region appeared in the SAXS profile, indicating the formation of TBA^+^-intercalated ColNFs. Above 60°C, the sample became strongly turbid, and needle-like crystals were observed by POM (Fig. 4C); many SAXS peaks appeared in the *q* range of 0.2 to 2 nm^−1^, which are attributed to the rectangular columnar superlattice with the lattice parameters *a* = 22.0 nm, *b* = 15.3 nm, and *c* = 2.84 nm (Fig. 4D). The lattice parameters *a* and *b* mostly match the long and short axes of the diamond-shape nanosheets, indicating that the ColNFs are packed as highly crystalline ColNF-Buns. Notably, the formation of these superstructures was totally reversible; the ColNF-Buns dissociated to mNSs by decreasing the temperature as shown in fig. S9. The structure formation with the increase in temperature observed here may be explained by dehydration of the hydrophobic TBA cations and/or decrease in relative permittivity of water. In either case, the nanosheet-nanosheet attraction increases although thermal diffusion increases (*45*). We suppose that the mNSs are rigidly arrested in the needle-like particle in the crystalline positions, considering the high structural regularity assured by the sharp SAXS peaks. It may be also possible that the mNSs are hopping among the columns as reported in other colloidal liquid crystal systems (*46*, *47*). We need further investigation with more precision techniques to clarify the details.

## DISCUSSION

The formation of the ColNFs and ColNF-Nem is basically explained by the classical DLVO and Onsager theories (*38*). In the mNS dispersions with low [TMA^+^], the negatively charged mNSs are surrounded by TMA^+^ ionic cloud so that the mNSs are stably dispersed because of electric double layer repulsions (*39*) as is explained by DLVO theory. With the increase in [TMA^+^], the electric double layer repulsion is screened because the diffuse layer thickness is inversely proportional to [TMA^+^]^0.5^. Increase in [TMA^+^] also causes TMA^+^ adsorption on the mNSs to partly cancel the negative charge of the mNSs. These situations lead to weak mNS-mNS attractions due to van der Waals forces and electrostatic attractions, rather than repulsions, so that mNSs are stacked to form the ColNFs. Because the attraction between the mNSs is not too strong, the mNSs can slide themselves to adjust the lateral position to the most stable one, the center of the 1D column. Then, the ColNFs form colloidal LC phase (ColNF-Nem) because free rotation of the ColNFs is restricted as the number and length of the ColNFs increase. According to Onsager’s theory, for a colloidal cylinder particle with the diameter *D* and the length *L*, a nematic phase appears above the volume fraction concentration φ_I_ = 3.3*v*_p_/*b*, where the excluded volume *b* = π*D*/4[*L*^2^ + *DL*(π + 3)/2 + π*D*^2^/4] and the volume of the particle *v*_p_ = π*D*^2^*L*/4. For the ColNF comprising *n* nanosheets stacked with the basal spacing of *d* in nanometers, the cylinder length *L* = *nd* so that φ_I_ is calculated as 4.7 vol % with *D* = 12 nm, *d* = 1.70 nm, and *n* = 1000. From this, [mNS] required for the ColNF-Nem formation is calculated as [mNS] = φ_I_*T*/*d* = 1.5 vol %, considering the nanosheet thickness *T* = 0.65 nm. This value is mostly in accordance with the experimental results shown in Fig. 1C. Note that if the mNSs are uniformly dispersed without forming ColNFs, then the theoretical [mNS] for LC phase formation is calculated as 20 vol %, which is much larger than the present systems. This again confirms that the mNSs were surely self-assembled into ColNFs in the present system.

In conclusion, the present strategy thus realized self-assemblies of the mNSs into 1D and hierarchical superstructures in the equilibrated solution phase. On the basis of the present fundamental findings, we are continuing research to obtain macroscopic scale materials such as superstructured composite fibers and vertically aligned nanosheet films suitable for electronics, optics, and catalysts. Formation of bulk/porous materials consist of the 1D nanofiber network structures is also expected. Considering the similarity of the present system with organic supramolecular polymers (*48*, *49*) and lyotropic chromonic liquid crystals (*50*, *51*), on-demand formation/decomposition and self-healing functionalities will be also expected, which have been generally difficult to attain with conventional inorganic materials.

## MATERIALS AND METHODS

### Materials

Titanium tetraisopropoxide [TIP; FUJIFILM Wako Pure Chemical (Wako), Osaka, Japan], tetramethylammonium hydroxide (TMAOH; 25 wt %; Wako), tetraethylammonium hydroxide [TEAOH; Tokyo Chemical Industry (TCI), Tokyo, Japan], tetrapropylammonium hydroxide (TPAOH; TCI), tetrabutylammonium hydroxide (TBAOH; TCI), and decyltrimethylammonium chloride (C_10_TMACl; TCI), and tris(2,2′-bipyridyl)ruthenium(II) chloride hexahydrate [Ru(bpy)_3_Cl_2_·6H_2_O; TCI] were used as received.

### Preparation of monodisperse nanosheet colloidal sol

To obtain mNS (0.52 vol %), 33.4 mmol (9.5 g) of TIP was dissolved in 150 ml of a TMAOH aqueous solution (0.273 M), followed by refluxing for 1 day, according to the method reported in the literature (*40*, *41*). TEAOH, TPAOH, or TBAOH was also used instead of TMAOH to obtain the sample with different counter cations of the mNS. The concentration of mNS and coexisting TxAOH was adjusted by evaporation and/or dilution with water or a TxAOH aqueous solution. The sample with lower [TMA^+^] was obtained by neutralizing the as-prepared sol to pH < 9.5 using Cl^−^-adsorbed anion-exchange resin, followed by dialysis. The samples added with EtOH were also prepared.

### Preparation of C_10_TMA^+^- and Ru(bpy)_3_^2+^-intercalated ColNF-Buns

C_10_TMACl solution (0.14 M) in EtOH/water mixture (70 wt % of EtOH) was added to the mNS dispersed in EtOH/water mixture (70 wt % of EtOH, [mNS] = 0.59 wt %, and [TMA] = 0.094 M). This mixture was then centrifuged (3000 rpm for 30 min), followed by removal of supernatant and addition of the EtOH/water mixture (70 wt % of EtOH). This process was repeated three times to lastly obtain the dispersion of C_10_TMA-intercalated ColNF or its bundles. Ru(bpy)_3_^2+^-intercalated ColNFs were obtained by adding mNS aqueous colloidal sol ([mNS] = 0.52 vol % and [TMA] = 0.273 M; 1 g) to the aqueous solution of Ru(bpy)_3_Cl_2_ (0.024 M; 1 g).

### Characterization

POM images were obtained with a BX51 microscope (Olympus, Tokyo, Japan). In the POM observation, λ plate (U-TP530, Olympus, Tokyo, Japan) with the retardation of 530 nm was used. A sample was sealed in a flat glass capillary (thickness of 0.2 mm) or a cell composed of two glass plates and a silicon rubber spacer (thickness of 0.5 mm). CLSM images were observed with A1R+ (Nikon, Tokyo, Japan) under scattering mode using the diode laser (wavelength is 405 nm). Fluorescence microscopy images were observed with a BZ-X800 of Keyence, Japan with the excitation wavelength of 470 ± 40 nm and the emission wavelength of 525 ± 50 nm. TEM images were obtained using a JEM-1400 and JEM-2100F operated at 200 kV (JEOL, Tokyo, Japan). The carbon 200 mesh–supported R2/1 Mo quantifoil (EM Japan, Tokyo, Japan) as grid for the cryo-TEM observation was subjected to a hydrophilic treatment with DII-29020HD (JEOL, Tokyo, Japan) for 20 s before use. Then, mNS aqueous colloidal sol containing ~1.5 M TMA^+^ and ~4 vol % of mNSs (Ic state) was dropped onto the grid at 25°C and 90% relative humidity in Leica EM GP (Leica Microsystems, Tokyo, Japan). Excess sample was removed by a filter paper, and the grids were quickly frozen in liquid ethane. The grids were observed in liquid nitrogen. Diluted Ru(bpy)_3_^2+^-intercalated ColNF colloidal sol was dropped onto the carbon supported Mo grids, and the grids were observed after vacuum-drying. SAXS measurements were performed on a Nanopix (Rigaku, Tokyo, Japan) with a CuKα radiation (40 mA and 30 kV). The camera length was set to 720 or 220 mm. Each sample for SAXS was sealed in a glass capillary with an inner diameter of 2.0 mm. The SAXS data were obtained as 2D images, and these were converted to intensity *I* versus scattering vector *q* format by circular averaging on the Igor Pro software. The dried powder of the nanosheet was measured with XRD on the XRD-7000L (Shimadzu Corporation, Kyoto, Japan) with a CuKα radiation (40 mA and 30 kV). The scan speed was 1.0°/min, the divergence slit was 1.0°, the divergence slit was 1.0°, and the receiving slit was 0.30 mm.

## Supplementary Materials

### This PDF file includes:

Figs. S1 to S9 Supplementary Notes for Figs. S4 and S5 References
