# Supplementary material for: Monodisperse nanosheet mesophases
Source: Sci Adv. 2024 Jun 5;10(23):eadk6452. doi: 10.1126/sciadv.adk6452 (PMC11152118; doi:10.1126/sciadv.adk6452)
Supplement: Supplementary file 1 — Figs. S1 to S9 Supplementary Notes for Figs. S4 and S5 References [file sciadv.adk6452_sm.pdf]

Science Advances
AAAS

Supplementary Materials for

Monodisperse nanosheet mesophases

Nobuyoshi Miyamoto *et al.*

Corresponding author: Nobuyoshi Miyamoto, miyamoto@fit.ac.jp; Takashi Kato, kato@chiral.t.u-tokyo.ac.jp

Sci. Adv. **10**, eadk6452 (2024)
DOI: 10.1126/sciadv.adk6452

This PDF file includes:

Figs. S1 to S9
Supplementary Notes for Figs. S4 and S5
References

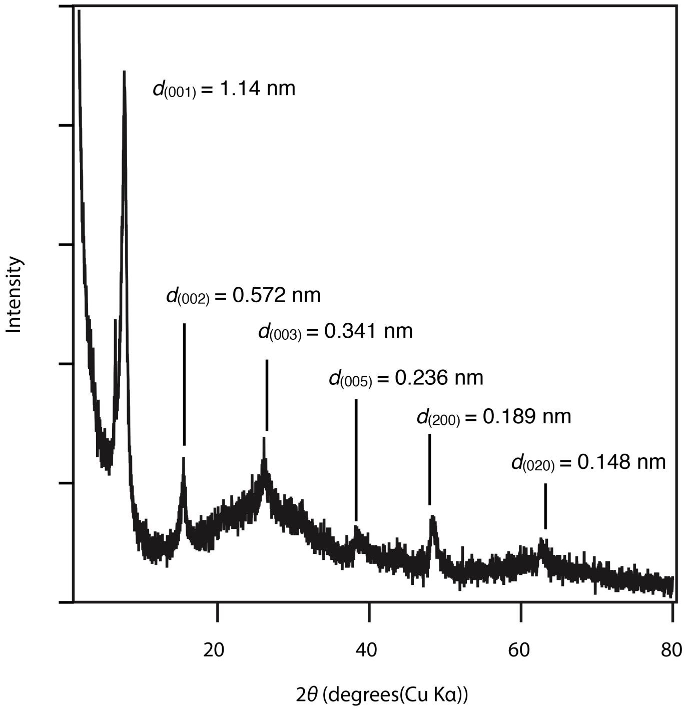

Fig. S1. Powder XRD pattern of the dried powder of the mNSs. In the XRD pattern, the peaks are observed at 2θ = 48.2° and 62.6° (d = 0.189 nm and 0.148 nm) which are ascribed to the in-plane (200) and (020) diffractions of the lepidocrocite-type layered titanate $[Ti_{1.825}O_4]^-$. In addition, peaks are also observed at 2θ = 7.74°, 15.5°, 26.1°, and 38.2° (d = 1.14, 0.572, 0.341, and 0.236 nm) which are due to the (001), (002), (003), and (005) diffractions of lamellar structure with the basal spacing of 1.14 nm. This basal spacing mostly corresponds to the sum of layer thickness (0.65 nm) and the diameter of TMA cation (0.4 nm), indicating that the nanosheets are restacked with interlayer TMA cations upon drying.

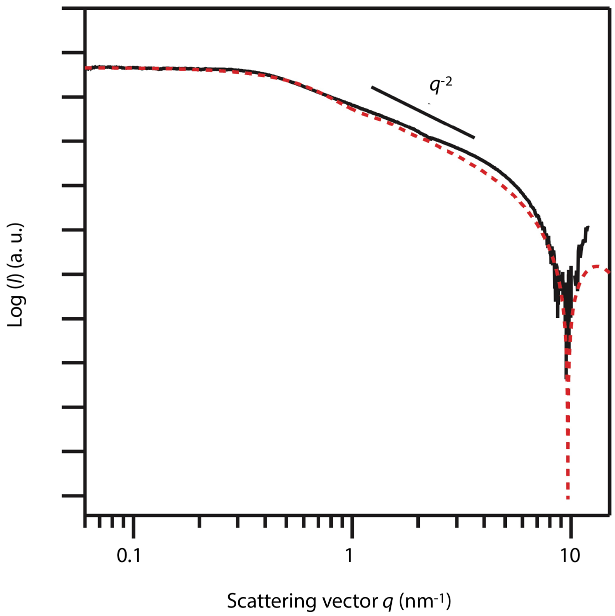

Fig. S2. SAXS pattern of the as-prepared mNSs dispersed in water. [TMA^+] = 0.27 M and [mNS] = 0.52 vol%. The red dashed line is the theoretical curve for a thin round disk with the diameter of 10 nm and the thickness of 0.65 nm.

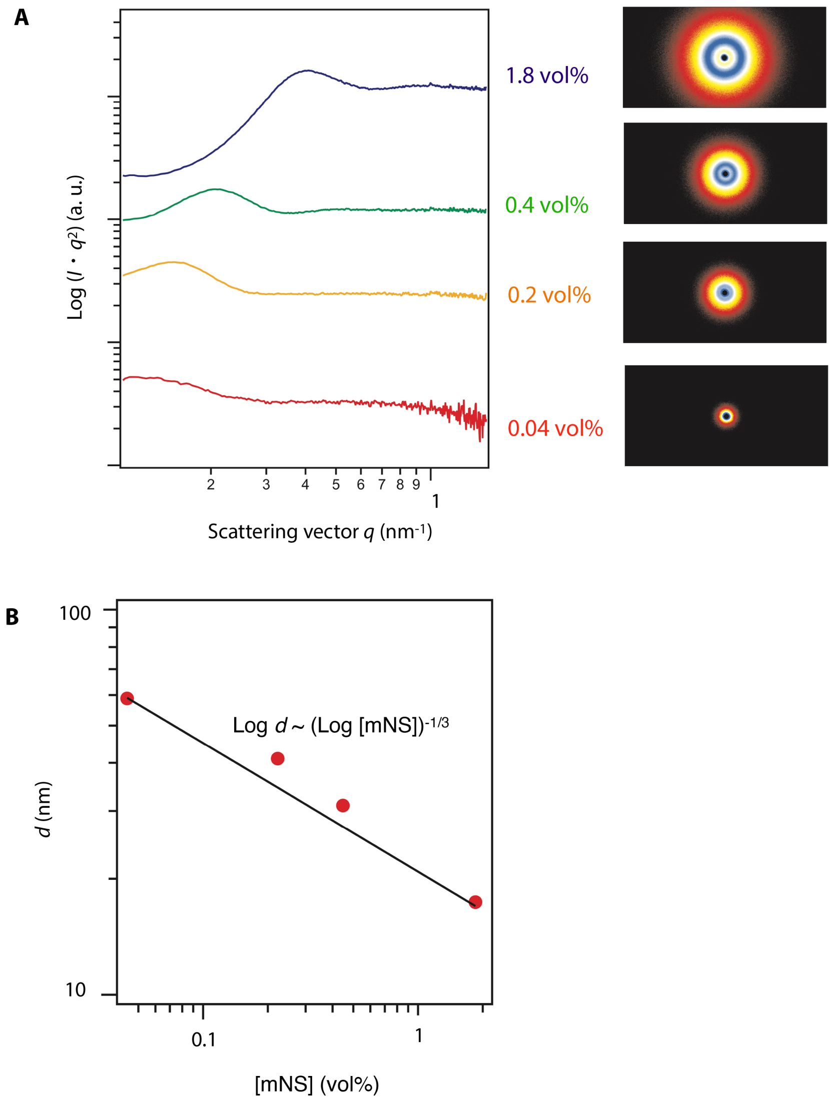

Fig. S3. SAXS analyses of the mNS aqueous dispersion ([TMA$^+$] = 10^{-3} M) with varied [mNS]. (**A**) SAXS profiles (I•q^2 vs q plots and 2D patterns). (**B**) The log-log plot of d-value of the broad peak in the SAXS profiles calculated as d=2π/q as the function of [mNS]. The broad peaks are due to the average distance between the nanosheets. As shown in (**B**), the log d is proportional to (log [mNS])$^{-1/3}$ so that we confirm the uniform and isotropic dispersion of the mNSs in this concentration range.

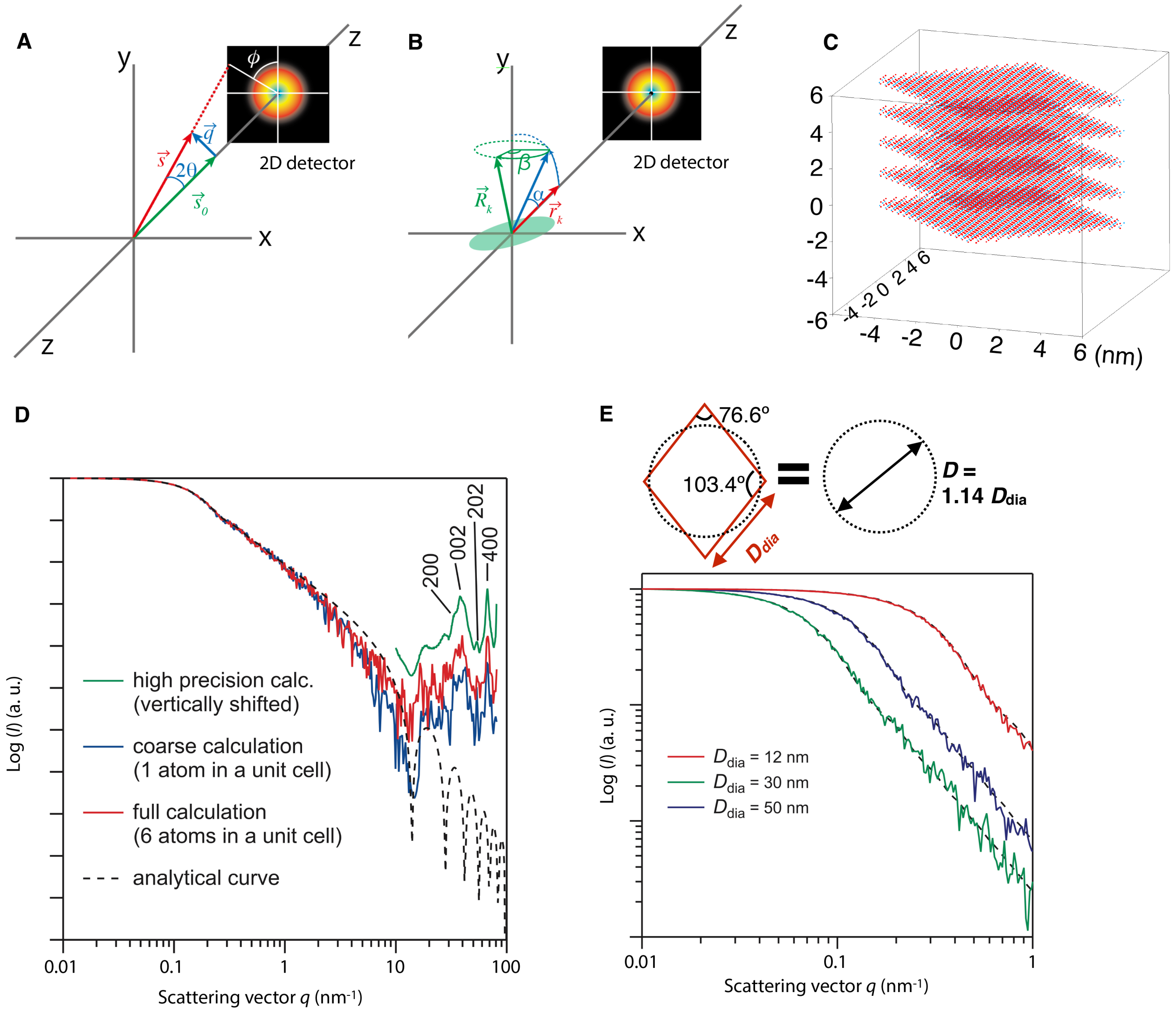

Fig. S4. SAXS profiles simulations (1). (**A**) (**B**) Geometries of the system, (**C**) The 3D plots of all the atoms in the 5-layer nanosheet stack used for the simulation, (**D**) Simulated and analytical SAXS curves of round-shaped nanosheets, (**E**) Simulated SAXS curves of diamond-shaped nanosheets (red, blue, and green lines) with the diameter D_{dia} and corresponding analytical curves for the round disks with the diameter D=1.14D_{dia}.

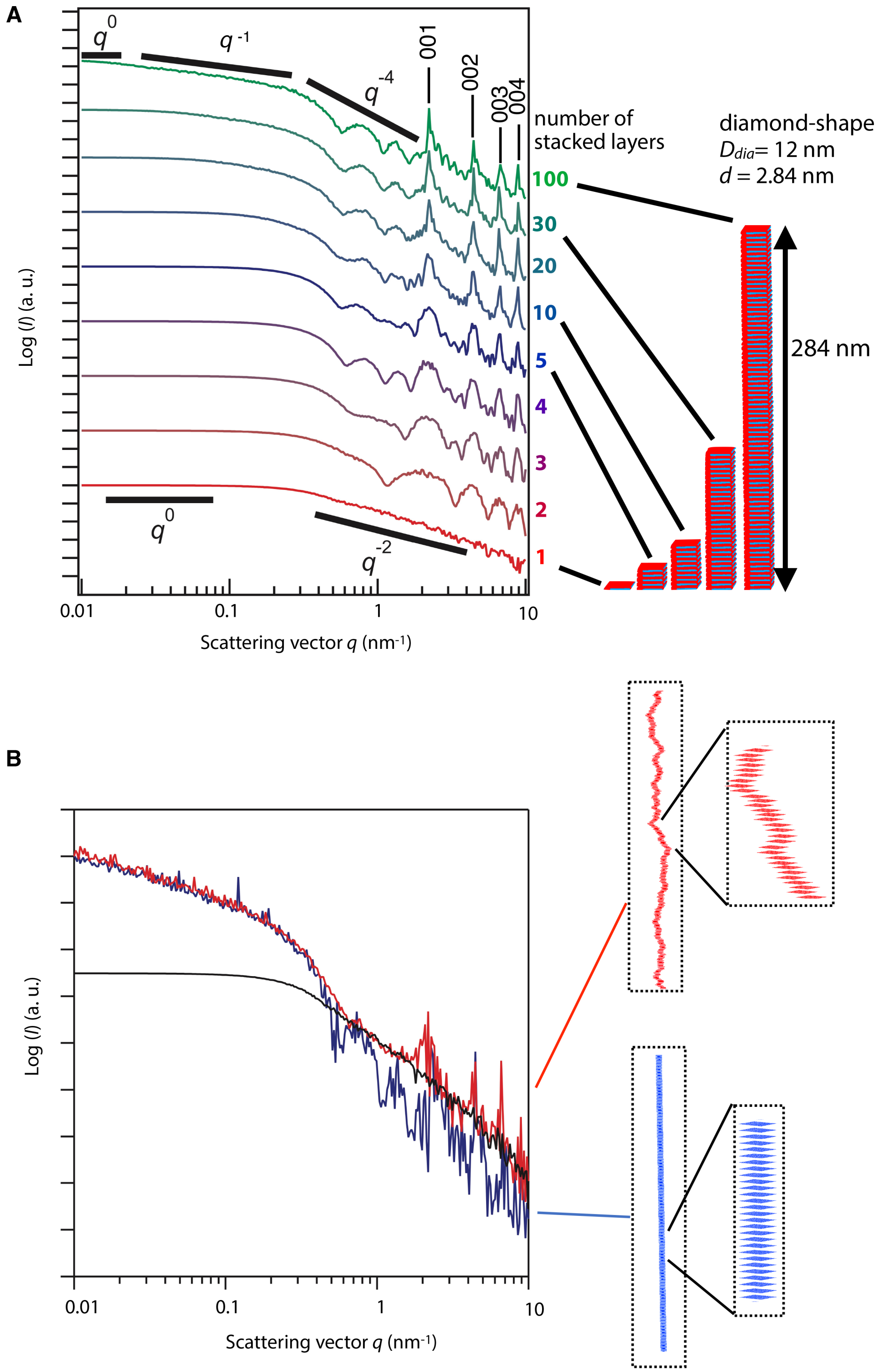

Fig. S5. SAXS profiles simulations (2). (**A**) Simulated and analytical SAXS curves of diamond-shaped mNSs with *n* stackings. (**B**) Comparison of the simulated SAXS curves for the mNSs forming straight stacking (blue line), stacking with undulation (red line), and no stacking (black line).

Supplementary notes for Fig. S4 and S5

We analyzed our SAXS data by comparing with simulated SAXS curves rather than by ordinary methods based on mathematical models because of the complexity of our superstructured system. In general, the scattering intensity $I(\vec{q})$ at a certain scattering vector $\vec{q}$ for an object with the volume V is principally calculated by integrating the scattering waves from all of the small volumes at the position $\vec{r}$ that have the electron density $\rho(\vec{r})$ as

$$I(\vec{q}) = \frac{F(\vec{q})F^*(\vec{q})}{V} \quad (1)$$

where

$$F(\vec{q}) = \int_V \rho(\vec{r})exp(\vec{q}\vec{r})d\vec{r} \quad (2)$$

Here, the scattering vector $\vec{q}$ is defined as

$$\vec{q} = \vec{s} - \overrightarrow{s_0}, |\vec{s}| = |\overrightarrow{s_0}| = 2\pi/\lambda \quad (3)$$

where $\vec{s}$ and $\overrightarrow{s_0}$ are the vectors of the incident and scattering beams with the wavelength λ, respectively. The magnitude of the scattering vector and the scattering angle 2θ is related as

$$q = |\vec{q}| = 4\pi sin\theta/\lambda \quad (4)$$

The overview of the system is shown in Fig. S4A.

In the present simulations, instead of the above integral that is usually solved by mathematical techniques, we simply summed up all or part of the atoms in the nanosheets or nanosheet superstructures, considering atomic scattering factor $f(\theta)$for O and Ti. Counter cations and solvents are not considered.

$$F(\vec{q}) \approx \sum_k f(\theta)exp(-i\vec{q}\overrightarrow{r_k}) \quad (5)$$

$$f_{Ti}(\theta) = 9.7597exp[-7.8508(sin\theta/\lambda)^2]$$
$$+7.3558exp[-0.5000(sin\theta/\lambda)^2]$$
$$+1.6991exp[-35.6338(sin\theta/\lambda)^2]$$
$$+1.9021exp[-116.1050(sin\theta/\lambda)^2] + 1.2807$$

$$f_{Ti}(\theta) = 3.0785exp[-13.2771(sin\theta/\lambda)^2]$$
$$+2.2868exp[-5.7011(sin\theta/\lambda)^2]$$
$$+1.5463exp[-0.3239(sin\theta/\lambda)^2]$$
$$+0.867exp[-32.9089(sin\theta/\lambda)^2] + 0.2508$$

To obtain the $I(q)$ *vs* q profile for an anisotropic object, averaging over the orientation angles α and β, as shown in Fig. S4B, was performed by repeating the calculation using uniform random numbers for α and β in the ranges of $-\pi/2 < \alpha < \pi/2$ and $-\pi < \beta < \pi$**.** After all, $F(q)$ is calculated according to the following triple summation:

$$F(q) = \frac{1}{N_{repeat}N_{\phi}N_{atoms}} \sum_{j=0}^{N_{repeat}} \sum_{k=0}^{N_{\phi}} \sum_{l=1}^{N_{atoms}} f(\theta)\, exp\left(-i\vec{q}\overrightarrow{R_l^{(l)}}\right) \quad (6)$$

where

$$\phi = \frac{k}{N_{\phi}}\pi$$

$$\vec{q} = (q\ sin\theta, q\ cos\theta sin\phi, q\ cos\theta sin\phi)$$

and $\overrightarrow{R_k^{(J)}}$ is the position vector after rotation of the original $\vec{r}$.

For the calculation, we set one nanosheet by placing Ti and O atoms inside the region with a diamond shape or a round shape, according to the crystal structure data (*52*). The nanosheets were then stacked with a certain periodicity. For example, Fig. S4C shows the 3D plot of the 5-layer stacks of the diamond-shaped nanosheets with D_{dia}=7.6 nm and stacking periodicity d = 1.7 nm that contain 1.5×10^4 atoms. In this case, the SAXS curve was obtained within 3 h on a MacPro (12-core Xeon 3.3GHz) with N_{repeat} = 2000, N_{Φ} = 100, and q division of 400.

To verify our calculation, we first calculated the SAXS curve for a round disk with the diameter D and the thickness L = 0.45 nm. We compared it with the corresponding analytically calculated theoretical curve according to the following equation:

$$I(q) = \frac{2}{q^2(D/2)^2}\left[1 - \frac{J_1[2q(D/2)]}{(q(D/2))}\right]\frac{sin^2(qL/2)}{(qL/2)^2} \qquad (7)$$

As expected, the simulated (red line in Fig. S4D) and analytical curves (dashed line in Fig. S4D) are mostly same, while we see some deviation at $q > 2$ nm^{-1} because the analytical curve is based on the uniform electron density distribution inside the disk, which is unrealistic. This deviation becomes slightly larger if only a part of atoms were considered for calculation (blue line in Fig. S4D). We also see the peaks due to in-plane crystalline structure when we perform high precision calculation. Thus, the calculation procedure is validated.

We then calculated the SAXS curve for the diamond shaped nanosheets with the edge length D_{dia} (Fig. S4E). The simulated curve is identical to the analytical curve for round disks with $D = 1.14D_{\mathrm{dia}}$. This result helps us to analyze the SAXS data for the irregular shaped nanosheet which is difficult to analyze with a mathematical model.

According to the methods above, we calculated the SAXS curves for the diamond-shaped monodisperse nanosheets that formed columnar nanofibers with the stacking number n = 1-100 (Fig. S5A). Single layer nanosheets (n = 1) showed q^{-2} slope at $q > 0.2$ nm^{-1}, whereas q^{-4} slope with waving emerged with the increase of n in this q-region. With $n > 10$ the slope of q^{-1} emerges at $q < 0.2$ nm^{-1}. As expected, these curves mostly coincide with an analytical form factor for rods, whereas deviation is large for smaller n. In addition to these scattering profiles, Bragg peaks due to the periodic stacking emerged at higher q region.

The present calculation method is very primitive; therefore, this is applicable for any irregular superstructures. In the Fig. S5B, the scattering curves of the columnar nanofibers with or without undulation are compared. As expected, the waving of the scattering curve disappeared if the columnar nanofibers had disordered shape.

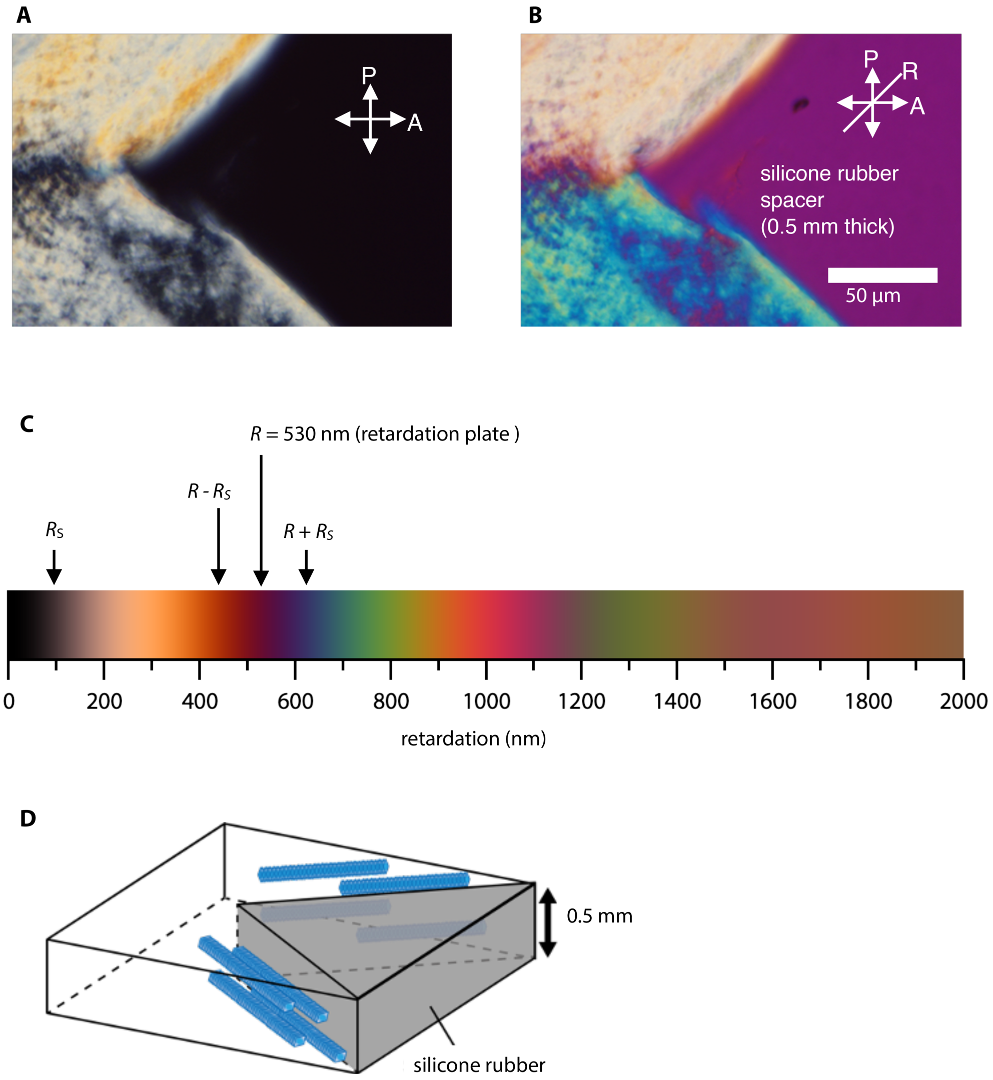

Fig. S6. Optional explanation for the POM observations: POM images of the ColNF-Nem sample ([mNS]= 3.1 vol% and [TMA^+] = 2.2 M) (A) without and (B) with the retardation plate, (C) a color chart showing the relationship between the optical retardation *R* and the interference color, and (D) a schematic drawing of this observation. The arrows labeled A and P are the directions of the analyzer and polarizer, respectively. The line labeled R is the direction of the retardation plate. In general, in a POM observation, we observe interference colors that correspond to the retardation *R* of the sample, which is the product of the birefringence index Δn and the thickness of the sample. The interference color is observable when the slow/fast axes (the axes with larger and smaller refractive index, respectively) of the birefringent sample is 45° to the directions of the polarizer/analyzer. In (**A**), we see a pale yellow color along the liquid/solid interface. This indicates that the nanosheet plane, which is parallel to the slow axis of the nanosheet, is preferentially aligned parallel or perpendicular to the interface that is set 45° to the polarizer/analyzer. However, we cannot determine whether the orientation direction is parallel or perpendicular from this observation. To identify the orientation direction, we inserted the retardation plate with the retardation *R* of 530 nm with its slow axis 45° to the polarizer/analyzer direction. As shown in (**B**), we observe a purple interference color in the spacer part, which has no

birefringence. In contrast, we observed yellow and blue interference colors at the spacer/liquid interface parallel and perpendicular to the retardation plate, respectively. These yellow and blue colors appear because the retardation of the sample R_s is added to or subtracted from that of the retardation plate (530 nm) when the slow axis of the sample is 0º or 90º, respectively, to the slow axis of the retardation plate. As obvious from the color chart (**C**), the interference colors for $R = 530 + R_s$ and $R = 530 - R_s$ are blue and yellow, respectively. Thus, from the image (**B**), we confirmed that the nanosheet plane is aligned perpendicular to the interface as schematically shown in (**D**).

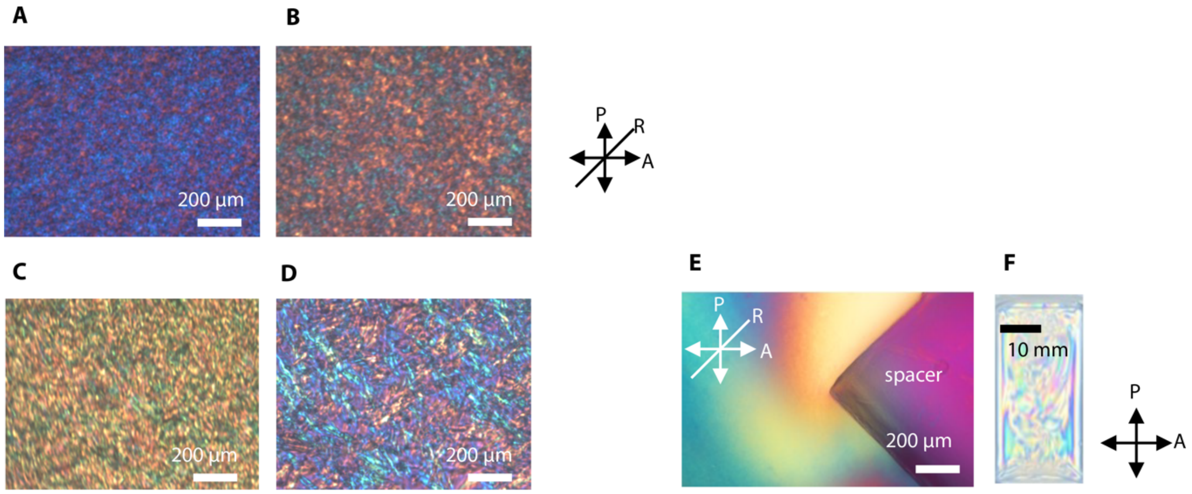

Fig. S7. Liquid crystallinity of the ColNFs dispersions intercalated with various cations. (**A**)-(**E**) POM and (**F**) macroscopic crossed-polarizer observations of the ColNFs intercalated with (**A**) TMA^+ (1.64 M) ([mNS]= 2.9 vol%), (**B**) TEA^+ (1.37 M) ([mNS]=2.5 vol%), (**C**) TPA^+ (1.37 M) ([mNS]=2.5 vol%), (**D**) TBA^+ (1.37 M) ([mNS]= 2.5 vol%), and (**E**) (**F**) $C_{10}TMA^+$ (2.3×10^{-2} M) with [mNS]=0.5 vol%. The arrows A and P show the directions of analyzer and paralyzer, respectively. The R is the direction of a λ plate with the retardation of 530 nm.

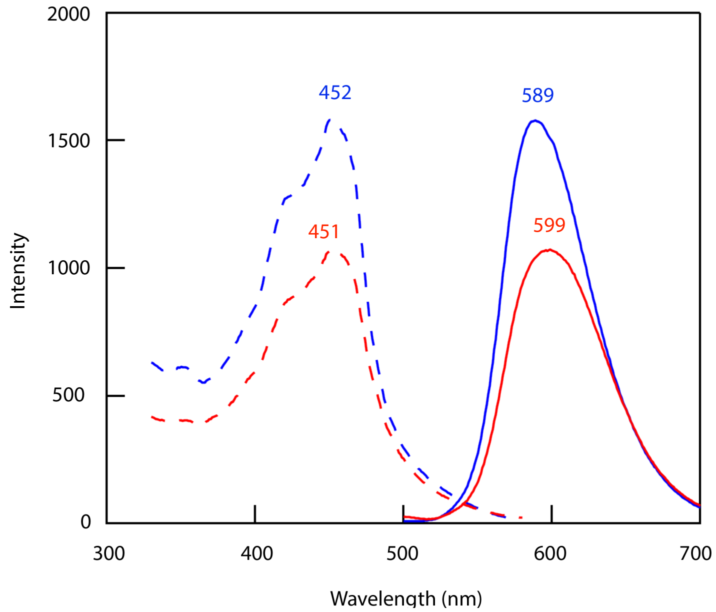

Fig. S8. Fluorescence spectra of the $Ru(bpy)_3^{2+}$ aqueous solution (blue lines) and the $Ru(bpy)_3^{2+}$ aqueous solution added with mNSs (red lines). [$Ru(bpy)_3^{2+}$] = 1.97×10^{-5} M and [mNS] = 5.56×10^{-4} vol%. The solid lines are the emission spectra obtained with the excitation at λ_{ex}= 452 nm and the dotted lines are the excitation spectra monitored at λ_{em} = 589 and 599 nm, respectively.

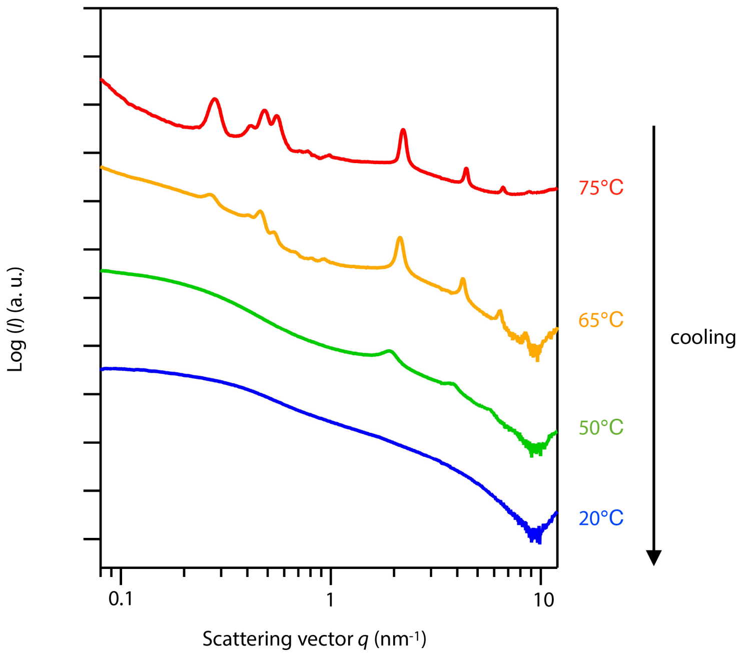

Fig. S9. **Temperature-controlled mesophase formation of the TBA^+/mNS aqueous colloid observed by SAXS in the cooling process.** [mNS] = 0.52 vol% and [TBA^+] = 0.27 M.

REFERENCES AND NOTES

1. I. Langmuir, The role of attractive and repulsive forces in the formation of tactoids, thixotropic gels, protein crystals and coacervates. *J. Chem. Phys.* **6**, 873–896 (1938).

2. F. M. van der Kooij, K. Kassapidou, H. N. W. Lekkerkerker, Liquid crystal phase transitions in suspensions of polydisperse plate-like particles. *Nature* **406**, 868–871 (2000).

3. J. C. P. Gabriel, F. Camerel, B. J. Lemaire, H. Desvaux, P. Davidson, P. Batail, Swollen liquid-crystalline lamellar phase based on extended solid-like sheets. *Nature* **413**, 504–508 (2001).

4. M. C. D. Mourad, J. E. G. J. Wijnhoven, D. D. van't Zand, D. van der Beek, H. N. W. Lekkerkerker, Gelation versus liquid crystal phase transitions in suspensions of plate-like particles. *Phil. Trans. R. Soc. A* **364**, 2807–2816 (2006).

5. N. Miyamoto, T. Nakato, Liquid crystalline nature of $K_4Nb_6O_{17}$ nanosheet sols and their macroscopic alignment. *Adv. Mater.* **14**, 1267–1270 (2002).

6. L. J. Michot, I. Bihannic, S. Maddi, S. S. Funari, C. Baravian, P. Levitz, P. Davidson, Liquid–crystalline aqueous clay suspensions. *Proc. Natl. Acad. Sci. U.S.A.* **103**, 16101–16104 (2006).

7. D. Sun, H.-J. Sue, Z. Cheng, Y. Martínez-Ratón, E. Velasco, Stable smectic phase in suspensions of polydisperse colloidal platelets with identical thickness. *Phys. Rev. E* **80**, 041704 (2009).

8. J. E. Kim, T. H. Han, S. H. Lee, J. Y. Kim, C. W. Ahn, J. M. Yun, S. O. Kim, Graphene oxide liquid crystals. *Angew. Chem. Int. Ed.* **50**, 3043–3047 (2011).

9. M. Nakayama, S. Kajiyama, T. Nishimura, T. Kato, Liquid-crystalline calcium carbonate: Biomimetic synthesis and alignment of nanorod calcite. *Chem. Sci.* **6**, 6230–6234 (2015).

10. M. Nakayama, S. Kajiyama, A. Kumamoto, T. Nishimura, Y. Ikuhara, M. Yamato, T. Kato, Stimuli-responsive hydroxyapatite liquid crystal with macroscopically controllable ordering and magneto-optical functions. *Nat. Commun.* **9**, 568 (2018).

11. J. Zhang, S. Uzun, S. Seyedin, P. A. Lynch, B. Akuzum, Z. Wang, S. Qin, M. Alhabeb, C. E. Shuck, W. Lei, E. C. Kumbur, W. Yang, X. Wang, G. Dion, J. M. Razal, Y. Gogotsi, Additive-free mxene liquid crystals and fibers. *ACS Cent. Sci.* **6**, 254–265 (2020).

12. W. Yang, S. Yamamoto, K. Sueyoshi, T. Inadomi, R. Kato, N. Miyamoto, Perovskite nanosheet hydrogels with mechanochromic structural color. *Angew. Chem. Int. Ed.* **60**, 8466–8471 (2021).

13. M. Nakayama, T. Kato, Biomineral-inspired colloidal liquid crystals: From assembly of hybrids comprising inorganic nanocrystals and organic polymer components to their functionalization. *Acc. Chem. Res.* **55**, 1796–1808 (2022).

14. S. Okamoto, S. Hachisu, On the long-range attractive force between plate-like gold particles: I. Fibrous aggregation. *J. Colloid Interface Sci.* **43**, 30–35 (1973).

15. M. B. J. Sigman, A. G. T. Hanrath, A. E. Saunders, F. Lee, B. A. Korgel, Solventless synthesis of monodisperse Cu_2S nanorods, nanodisks, and nanoplatelets. *J. Am. Chem. Soc.* **125**, 16050–16057 (2003).

16. K. L. Young, M. R. Jones, J. Zhang, R. J. Macfarlane, R. Esquivel-Sirvent, R. J. Nap, J. Wu, G. C. Schatz, B. Lee, C. A. Mirkin, Assembly of reconfigurable one-dimensional colloidal superlattices due to a synergy of fundamental nanoscale forces. *Proc. Natl. Acad. Sci. U.S.A.* **109**, 2240–2245 (2012).

17. J. Zhang, T. Vad, M. Heidelmann, T. E. Weirich, W. F. Sager, Self-assembly of biaxial discorectangular lead carbonate nanosheets into stacked ribbons studied by SAXS and HAADF-STEM tomographic tilt series. *Soft Matter* **10**, 9511–9522 (2014).

18. S. Jana, P. Davidson, B. Abecassis, CdSe nanoplatelets: Living polymers. *Angew. Chem. Int. Ed.* **55**, 9371–9374 (2016).

19. H. Shan, L. Liu, J. He, Q. Zhang, W. Chen, R. Feng, C. Chang, P. Zhang, P. Tao, C. Song, W. Shang, T. Deng, J. Wu, Controllable assembly of Pd nanosheets: A solution for 2D materials storage. *CrstEngComm* **19**, 3439–3444 (2017).

20. S. Jana, M. D. Frutos, P. Davidson, B. Abecassis, Ligand-induced twisting of nanoplatelets and their self-assembly into chiral ribbons. *Sci. Adv.* **3**, e1701483 (2017).

21. J. Yu, M. Sharma, Y. Wang, S. Delikanli, H. D. Baruj, A. Sharma, H. V. Demir, C. Dang, Modulating emission properties in a host–guest colloidal quantum well superlattice. *Adv. Opt. Mater.* **10**, 2101756 (2021).

22. T. Paik, D.-K. Ko, T. R. Gordon, V. Doan-Nguyen, C. B. Murray, Studies of liquid crystalline self-assembly of GdF_3 nanoplates by in-plane, out-of-plane SAXS. *ACS Nano* **5**, 8322–8330 (2011).

23. X. Ye, J. Chen, M. Engel, J. A. Millan, W. Li, L. Qi, G, Xing, J. E. Collins, C. R. Kagan, J. Li, S. C. Glotzer, C. B. Murray, Competition of shape and interaction patchiness for self-assembling nanoplates. *Nat. Chem.* **5**, 466–473 (2013).

24. R. Momper, H. Zhang, S. Chen, H. Halim, E. Johannes, S. Yordanov, D. Braga, B. Blulle, D. Doblas, T. Kraus, M. Bonn, H. I. Wang, A. Riedinger, Kinetic control over self-assembly of semiconductor nanoplatelets. *Nano Lett.* **20**, 4102–4110 (2020).

25. X. Liu, Z. Chen, Q. Liu, G. H. Sheetah, N. Sun, P. Zhao, Y. Xie, I. I. Smalyukh, Morphological and orientational controls of self-assembly of gold nanorods directed by evaporative microflows. *ACS Appl. Mater. Interfaces* **13**, 53143–53154 (2021).

26. A. E. Saunders, A. Ghezelbash, D.-M. Smilgies, B. Michael, J. Sigman, B. A. Korgel, Columnar self-assembly of colloidal nanodisks. *Nano Lett.* **6**, 2959–2963 (2006).

27. I. Cherniukh, G. Raino, T. V. Sekh, C. Zhu, Y. Shynkarenko, R. A. John, E. Kobiyama, R. F. Mahrt, T. Stoferle, R. Erni, M. V. Kovalenko, M. I. Bodnarchuk, Shape-directed co-assembly of lead halide perovskite nanocubes with dielectric nanodisks into binary nanocrystal superlattices. *ACS Nano* **15**, 16488–16500 (2021).

28. K. Deng, X. Huang, Y. Liu, L. Xu, R. Li, J. Tang, Q. L. Lei, R. Ni, C. Li, Y. S. Zhao, H. Xu, Z. Wang, Z. Quan, Supercrystallographic reconstruction of 3D nanorod assembly with collectively anisotropic upconversion fluorescence. *Nano Lett.* **20**, 7367–7374 (2020).

29. K. Sano, Y. S. Kim, Y. Ishida, Y. Ebina, T. Sasaki, T. Hikima, T. Aida, Photonic water dynamically responsive to external stimuli. *Nat. Commun.* **7**, 12559 (2016).

30. K. Deng, Z. Luo, L. Tan, Z. Quan, Self-assembly of anisotropic nanoparticles into functional superstructures. *Chem. Soc. Rev.* **49**, 6002–6038 (2020).

31. J. Uchida, B. Soberats, M. Gupta, T. Kato, Advanced functional liquid crystals. *Adv. Mater.* **34**, 2109063 (2022).

32. B. T. Diroll, B. Guzelturk, H. Po, C. Dabard, N. Fu, L. Makke, E. Lhuillier, S. Ithurria, 2D II-VI semiconductor nanoplatelets: From material synthesis to optoelectronic integration. *Chem. Rev.* **123**, 3543–3624 (2023).

33. D. Nepal, S. Kang, K. M. Adstedt, K. Kanhaiya, M. R. Bockstaller, L. C. Brinson, M. J. Buehler, P. V. Coveney, K. Dayal, J. A. El-Awady, L. C. Henderson, D. L. Kaplan, S. Keten, N. A. Kotov, G. C. Schatz, S. Vignolini, F. Vollrath, Y. Wang, B. I. Yakobson, V. Tsukruk, H. Heinz, Hierarchically structured bioinspired nanocomposites. *Nat. Mater.* **22**, 18–35 (2023).

34. T. Kato, M. Yoshio, T. Ichikawa, B. Soberats, H. Ohno, M. Funahashi, Transport of ions and electrons in nanostructured liquid crystals. *Nat. Rev. Mater.* **2**, 17001 (2017).

35. N. Miyamoto, Y. Ohsedo, T. Nakato, Colloidal nanosheets, in *Inorganic Nanosheets and Nanosheet-Based Materials* (Springer Japan, 2017), pp. 201–260.

36. M. Osada, T. Sasaki, The rise of 2D dielectrics/ferroelectrics. *APL Mater.* **7**, 120902 (2019).

37. T. Taniguchi, L. Nurdiwijayanto, R. Ma, T. Sasaki, Chemically exfoliated inorganic nanosheets for nanoelectronics. *Appl. Phys. Rev.* **9**, 021313 (2022).

38. L. Onsager, The effects of shape on the interaction of colloidal particles. *Ann. N. Y. Acad. Sci.* **51**, 627–659 (1949).

39. F. Geng, R. Ma, A. Nakamura, K. Akatsuka, Y. Ebina, Y. Yamauchi, N. Miyamoto, Y. Tateyama, T. Sasaki, Unusually stable ∼100-fold reversible and instantaneous swelling of inorganic layered materials. *Nat. Commun.* **4**, 1632 (2013).

40. T. Ohya, A. Nakayama, T. Ban, Y. Ohya, Y. Takahashi, Synthesis and characterization of halogen-free, transparent, aqueous colloidal titanate solutions from titanium alkoxide. *Chem. Mater.* **14**, 3082–3089 (2002).

41. E. L. Tae, K. E. Lee, J. S. Jeong, K. B. Yoon, Synthesis of diamond-shape titanate molecular sheets with different sizes and realization of quantum confinement effect during dimensionality reduction from two to zero. *J. Am. Chem. Soc.* **130**, 6534–6543 (2008).

42. T. Sasaki, M. Watanabe, Osmotic swelling to exfoliation. Exceptionally high degrees of hydration of a layered titanate. *J. Am. Chem. Soc.* **120**, 4682–4689 (1998).

43. K. Kanie, T. Yasuda, M. Nishii, S. Ujiie, T. Kato, Hydrogen-bonded lyotropic liquid crystals of folic acids: Responses to environment by exhibiting different complex patterns. *Chem. Lett.* **30**, 480–481 (2001).

44. M. Ogawa, M. Tsujimura, K. Kuroda, Incorporation of tris(2,2′-bipyridine)ruthenium(II) in a synthetic swelling mica with poly(vinylpyrrolidone). *Langmuir* **16**, 4202–4206 (2000).

45. K. Kamada, N. Soh, Temperature-controlled reversible exfoliation-stacking of titanate nanosheets in an aqueous solution containing tetraalkylammonium ions. *RSC Adv.* **4**, 8682–8685 (2014).

46. H. Mundoor, J. S. Wu, H. H. Wensink, I. I. Smalyukh, Thermally reconfigurable monoclinic nematic colloidal fluids. *Nature* **590**, 268–274 (2021).

47. L. Alvarez, M. P. Lettinga, E. Grelet, Fast diffusion of long guest rods in a lamellar phase of short host particles. *Phys. Rev. Lett.* **118**, 178002 (2017).

48. L. Brunsveld, B. J. B. Folmer, E. W. Meijer, R. P. Sijbesma, Supramolecular polymers. *Chem. Rev.* **101**, 4071–4098 (2001).

49. T. Kato, Self-assembly of phase-segregated liquid crystal structures. *Science* **295**, 2414–2418 (2002).

50. J. Lydon, Chromonic review. *J. Mater. Chem.* **20**, 10071–10099 (2010).

51. H.-S. Park, O. D. Lavrentovich, Lyotropic chromonic liquid crystals: Emerging applications, in *Liquid Crystals Beyond Displays* (Wiley, 2012), pp. 449–484.

52. I. E. Grey, C. Li, I. C. Madsen, J. A. Watts, The stability and structure of $Cs_x[Ti_{2-x/4}\square_{x/4}]O_4$, $0.61 < x < 0.65$. *J. Solid State Chem.* **66**, 7–19 (1987).
